# Combining microscopy with mesoscopy using optical and optoacoustic label-free modes

**DOI:** 10.1038/srep12902

**Published:** 2015-08-26

**Authors:** Dominik Soliman, George J. Tserevelakis, Murad Omar, Vasilis Ntziachristos

**Affiliations:** 1 Institute of Biological and Medical Imaging, Helmholtz Zentrum München, Ingolstädter Landstr. 1, 85764 Neuherberg, Germany; Chair for Biological Imaging, Technische Universität München, Ismaningerstr. 22, 81675 München, Germany.

## Abstract

Biology requires observations at multiple geometrical scales, a feature that is not typically offered by a single imaging modality. We developed a hybrid optical system that not only provides different contrast modes but also offers imaging at different geometrical scales, achieving uniquely broad resolution and a 1000-fold volume sampling increase compared to volumes scanned by optical microscopy. The system combines optoacoustic mesoscopy, optoacoustic microscopy and two-photon microscopy, the latter integrating second and third harmonic generation modes. Label-free imaging of a mouse ear and zebrafish larva *ex-vivo* demonstrates the contrast and scale complementarity provided by the hybrid system. We showcase the superior anatomical orientation offered by the label-free capacity and hybrid operation, over fluorescence microscopy, and the dynamic selection between field of view and resolution achieved, leading to new possibilities in biological visualization.

Optical microscopy plays a fundamental role in studying structural and functional information at the cellular and sub-cellular level, as well as monitoring cell organization and formation into organs^1^. However, photon scattering in tissues does not allow for microscopic imaging at depths exceeding a few hundred micrometers *in vivo*. This limitation prohibits whole-body or organ level optical microscopy studies in small animals and has driven the development of mesoscopic optical techniques that can scan larger fields of view and depths^2^. Imaging of larger fields of view in transparent specimens has been achieved by optical projection tomography (OPT)^3^ and selective plane illumination microscopy (SPIM)^4^. Mesoscopic imaging of non-transparent specimens has been also demonstrated by mesoscopic fluorescence tomography (MFT)^5^ and mesoscopic optoacoustic imaging^6^,^7^,^8^. We have recently introduced raster-scan optoacoustic mesoscopy (RSOM), which achieves an axial resolution of 4 μm and a lateral resolution of ~20 μm up to a depth of 3 mm and 7 μm axial/~30 μm lateral resolution at depths of 5 mm^9^. However, none of these approaches achieve concurrently large penetration depths and microscopic resolution imaging in large, non-transparent specimens.

However, for many biological applications, such as monitoring of developmental mechanisms or understanding the spatial heterogeneity of disease, it is necessary to simultaneously capture both, cellular and whole-organism level processes, as well as their mutual interactions^1^. There have been many approaches that attempt to combine different contrast mechanisms in microscopy. Optoacoustic microscopy in particular has been combined with second harmonic generation (SHG) microscopy^10^, two-photon and confocal fluorescence microscopy^11^, optical coherence tomography (OCT)^12^, pulse-echo ultrasound imaging^13^ and single-photon excitation autofluorescence imaging^14^. However, hybrid implementations achieving multi-scale optical imaging have not been common. Emerging optoacoustic systems, such as RSOM, have some intrinsic multi-scale capabilities by detecting different frequencies^9^ or combining optical and optoacoustic components^15^ but they operate with a single field of view and do not achieve optical-diffraction limited resolution, a prerequisite for cellular and sub-cellular imaging.

In this work, we investigated the possibility of scanning volumes that are 10^3^ times larger than those conventionally scanned by optical microscopy and achieving *zoom-in* ability with optical-diffraction limited resolution. To achieve this level of scalability in three dimensions, we researched the combination of RSOM with optical and optoacoustic microscopy, integrated into one hybrid device and a common coordinate system. The final integrated multi-photon and multi-scale optoacoustic microscope (IMMSOM) combines RSOM with optical-resolution optoacoustic microscopy (OM), two-photon excitation fluorescence (TPEF), second harmonic generation (SHG) and third harmonic generation (THG) microscopy (see Fig. 1). We interrogated label-free imaging abilities for all modalities employed in the hybrid system and further demonstrate biological imaging capacities based on intrinsic tissue contrast. The advantages of label-free imaging were particularly researched in the context of the combination of optoacoustic mesoscopy and microscopy, imaging the same anatomical markers so that an accurate orientation and image co-registration between the different scales could be achieved, further integrating the other microscopy modes. This is a critical parameter of the system designed, as it then allows for an accurate relation of optical labels and non-linear signals at optical-diffraction limited resolution to the mesoscopy scan, achieved with acoustical-diffraction limited resolution.

## Results

### Experimental setup

**Figure 2:**
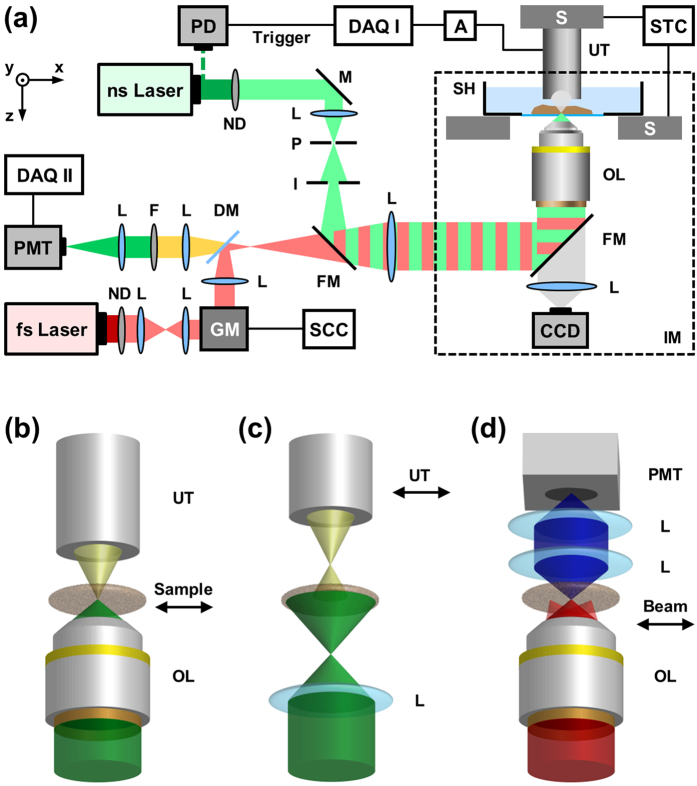
Experimental setup. (**a**) Scheme of the IMMSOM system in the OM configuration. The different scanning modes are illustrated for (**b**) OM, (**c**) RSOM and (**d**) THG measurements. The respective part that is moved for scanning is indicated by the arrows. Abbreviations: A, amplifier; DAQ, data acquisition card; DM, dichroic mirror; F, optical filter; FM, flip mount mirror; GM, galvanometric mirrors; I, iris aperture; IM, inverted microscope; L, lens; M, mirror; ND, neutral density filter; OL, objective lens; P, pinhole; PD, photo diode; PMT, photomultiplier tube; S, motorized xyz-stage; SCC, scan control; SH, sample holder; STC, stage control; UT, ultrasound transducer.

The multi-scale hybrid imaging system (Fig. 2, Methods section) employs two different laser sources for the optoacoustic and non-linear modalities, respectively, which are coupled into an inverted microscope. Both multi-photon and optoacoustic microscopy utilize focused light illumination, whereas RSOM employs broad illumination, typically over a several millimeter radius circular pattern.

### Spatial resolution characterization

**Figure 3:**
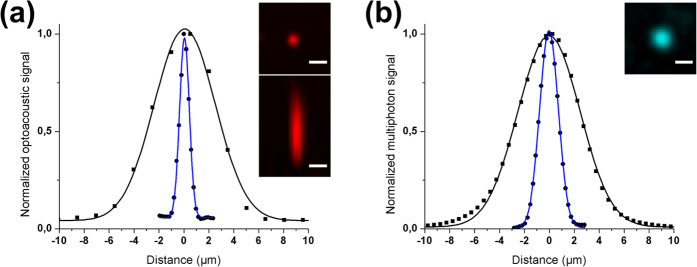
Spatial resolution characterization of the IMMSOM microscopy modalities. (**a**) OM measurement of a black 954 nm microsphere. The insets show MIPs of the imaged sphere in lateral (top) and axial (bottom) view. Gaussian fitted profile plots are illustrated by the blue (lateral) and black (axial) curves. (**b**) Measurements of a 100 nm fluorescent nanobead with TPEF and a glass-air optical interface with THG. The inset shows the lateral view of the nanobead. Blue and black curves represent the Gaussian fitted profiles of the sphere (lateral, TPEF) and the glass-air interface (axial, THG), respectively. Scale bars: 2 μm.

In order to characterize the spatial resolution of the optoacoustic microscopy modality, we measured black polystyrene microspheres with 954 nm diameter. An image of a single sphere was obtained by scanning an area of 8 μm × 8 μm in 0.2 μm steps. Figure 3(a) shows the Gaussian fitted lateral (blue line, R^2^ = 0.997) and axial (black line, R^2^ = 0.993) profile plots of the imaged microsphere. Maximum intensity projections (MIPs) in lateral and axial view are depicted by the upper and lower inset, respectively. The full width at half maximum (FWHM) of the lateral profile was 910 nm, corresponding to an estimated lateral resolution of 829 nm (see Methods section). The observed deviation from the FWHM of an ideal Airy disk (584 nm) was probably caused by an imperfect laser beam quality and optical aberrations. While the lateral resolution of the optoacoustic microscopy system is determined by the focusing capability of the objective lens, the axial resolution is governed by the detection bandwidth of the transducer^16^. It was found to be 5.78 μm as defined by the FWHM of the axial profile of the microsphere. On the other hand, the spatial resolution of the RSOM modality depends solely on the characteristics of the employed transducer, since the illuminated area is much broader than its acoustic focus. It was shown to be ~30 μm laterally and ~7 μm axially up to a depth of 5 mm in a previous work^17^.

Regarding the spatial resolution characterization of the multi-photon modalities, we followed two distinct methodologies for the precise determination of the minimum detail that can be resolved by the developed system in the lateral and the axial direction. More specifically, the lateral resolution was estimated through the TPEF imaging of 100 nm fluorescent spheres, while the axial resolution was experimentally determined by measuring the THG profile at the glass-air interface of a standard 170 μm coverslip. Figure 3(b) shows the lateral profile plot of a fluorescent nanosphere as depicted by the inset, fitted with a Gaussian curve (blue line, R^2^ = 0.999). It yielded a FWHM of 1.75 μm, which corresponds to the estimated lateral resolution. The deviation from the diffraction limited spot size of 1.18 μm can be justified primarily by uncompensated optical aberrations of the system. The second plot in Fig. 3(b) illustrates the axial THG spot measurements. The Gaussian fit (black line, R^2^ = 0.998) yielded a FWHM of 5.84 μm. The latter value stands by definition for the measured confocal parameter (depth of focus), since it is proven that in the case of tight focusing, both the excitation and the Harmonics beams are characterized by the same axial extent^18^.

### Hybrid mouse ear imaging

**Figure 4:**
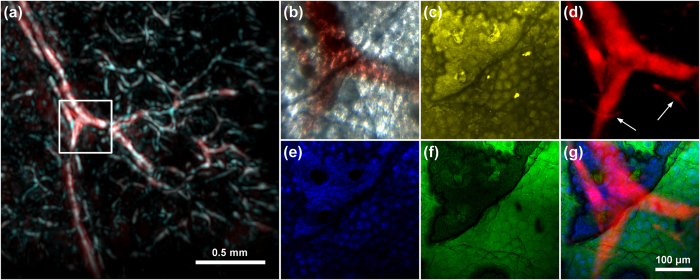
Hybrid label-free imaging of a mouse ear *ex-vivo*. (**a**) RSOM image of a larger region showing an overlay of the full detection bandwidth (red) and high frequencies (cyan) images. The white box indicates the region of microscopy scans with the (**b**) brightfield, (**c**) TPEF, (**d**) OM, (**e**) SHG and (**f**) THG modalities. (**g**) Overlay of the OM, SHG and THG images.

To demonstrate the multi-scale capabilities of the developed IMMSOM system, we initially imaged an excised mouse ear. As a first step, we performed a measurement with the RSOM modality, scanning a 2 mm × 2 mm region of the specimen. An image was generated by taking the MIP of the absolute value of the reconstruction volume along a depth of ~0.4 mm, corresponding to the thickness of the ear. Figure 4(a) illustrates an overlay of the full detection bandwidth (red color) and the high-frequency (cyan color) images of the mouse ear, showing blood vessels of different sizes. Since smaller structures generally generate higher frequencies than bigger ones, the high-frequency image primarily contains high-resolution features. After the mesoscopy measurement, a small region of 375 μm × 375 μm (indicated by the white box) at the bifurcation of one of the bigger vessels was selected for subsequent high-resolution microscopy imaging. Figure 4(d) represents the MIP of the optoacoustic microscopy measurement, showing the branching of the big vessel with high resolution as well as smaller features (highlighted by the white arrows) otherwise blurred or invisible through RSOM. Furthermore, a contrast variation across the large imaged vessel is observed.

Next, the same region was imaged simultaneously with the SHG and THG modalities. The images were recorded through a depth of 50 μm. MIPs of the volume scanned by SHG and THG microscopy are illustrated in Fig. 4(e) and Fig. 4(f), respectively. The THG signals were obtained mainly at the optical interface between the coverslip glass and the outermost epidermis layer of the mouse ear skin. This stratified squamous epithelium is known to be predominantly consisting of proliferating and differentiating keratinocytes, whose structure is clearly visible in Fig. 4(f). The mean diameter of these cells is in the order of 25 μm. Through THG imaging, we can additionally distinguish a large area characterized by the absence of cells and minimum signal generation. Within this region, it is also observed that there are three well-defined sub-regions, most likely to be hair follicles. On the other hand, SHG imaging shows the collagen constituting the underlying dermis layer of the skin^19^. Due to the relatively small thickness of the examined specimen, the collagen layer seems to follow the keratinocytes pattern across the region densely populated by cells. However, within the cell-free area, the obtained contrast is adequate enough to reveal the structure and the internal distribution of the respective collagen fibrils. Afterwards, the volume was scanned a second time to record the TPEF resulting from the autofluorescence arising mainly from the elastin^19^, which constitutes a basic component of the extracellular matrix in the dermis layer (Fig. 4(c)). Even though in this case, the TPEF image is quite similar to the respective SHG distribution, we can observe a pronounced signal difference in the three hair follicles regions, indicating the absence of any collagen quantity within them. Finally, a brightfield image of the examined region was recorded with the CCD camera for comparison, shown in Fig. 4(b). An overlay of the OM, SHG and THG images is shown in Fig. 4(g).

### Hybrid zebrafish larva imaging

**Figure 5:**
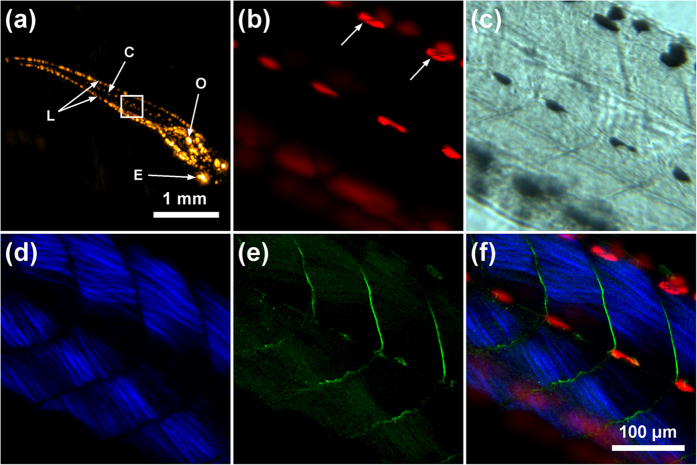
Hybrid label-free imaging of a wildtype zebrafish larva *ex-vivo*. (**a**) RSOM image of the zebrafish, visualizing lateral (L) and central (C) melanocyte stripes, inner organs (O) and the eyes (E). Microscopy images were obtained from the area indicated by the white box. Imaging was performed with the (**b**) OM, (**c**) brightfield, (**d**) SHG and (**e**) THG modalities. (**f**) Overlay of the OM, SHG and THG images.

To further investigate the performance of IMMSOM on another specimen, we additionally imaged a 6-days-old wildtype zebrafish larva *ex-vivo*, measuring a length of ~4.5 mm and a diameter of ~0.6 mm. We followed a similar procedure as described for the mouse ear measurement. The RSOM image, representing the full detection bandwidth for a scanning region of 4 mm × 4 mm, is depicted in Fig. 5(a). It shows the typical lateral (L) and central (C) pigment stripes along the fish body. Furthermore, additional anatomical features such as the eyes (E) and inner organs (O) can be identified^9^. Subsequent microscopy scans were performed on a selected region of 315 μm × 315 μm (indicated by the white box) at the central body region of the larva. Figure 5(b) illustrates the OM image where melanocytes around a central plane inside the fish body are clearly visible. When compared to the brightfield image (Fig. 5(c)) at the same depth inside the body, the OM image provides better contrast variations within the melanocytes (indicated by the white arrows). The blurring of the lower row of melanocytes originates from their position outside of the illumination focus, which had a confocal parameter in the order of a few μm.

The simultaneous SHG and THG imaging depicted in Fig. 5(d),(e), respectively, revealed the muscular structure of the larva body at the same focal plane as the performed OM measurement. In the SHG image, the different muscle segments (myomeres) constituting the musculature of the fish body can be distinguished. The spatial resolution of the modality is sufficient to resolve single muscle fibrils, which acted as the main source of SHG signals^20^ due to their pronounced anisotropic optical properties^21^. Furthermore, the THG signals originated mainly from the regions between the myomeres, most probably representing vertical myosepta; thin sheets of connective tissue that separate and support the myomeres and to which the myofibrils are attached. Finally, Fig. 5(f) shows a triple-modal image as the overlay of OM, SHG and THG signals.

## Discussion

We developed a hybrid optical and optoacoustic imaging system (IMMSOM) achieving a uniquely broad resolution and depth penetration range by combining optical and optoacoustic mesoscopy and microscopy techniques into a single device. By employing label-free multi-contrast imaging, the system demonstrated potential for the simultaneous visualization of different anatomical features in biological organisms without the need of external labels. The system performance at different scales was highlighted through the imaging of large specimens, demonstrating the extended field of view using RSOM and the zoom-in capabilities based on optical and optoacoustic microscopy. By using the same contrast, RSOM and optoacoustic microscopy can be seamlessly combined, providing then an accurate coordinate frame for the additional registration of the multi-photon images. In that respect, the hybrid system demonstrated a first advantage of enabling better orientation during scanning and the potential for the dynamic selection of the region, or regions of interest to be imaged based on the original RSOM assessment of the sample.

Through hybrid imaging of a mouse ear, we demonstrated that a general view of the ear vasculature over an extended 2 mm × 2 mm field of view can be extracted through the entire ear thickness. The inherent scalability of this modality enabled a concurrent visualization of bigger and smaller vessels by the separate processing of high frequencies, the latter generated by smaller features and normally masked by low-frequency signals. Then, we demonstrated the zoom-in capability of IMMSOM by subsequent high-resolution imaging of a selected region around a prominent vessel branching. Moreover, label-free second and third harmonic imaging revealed fundamental skin and tissue structure and components (vessels, collagen, elastin and keratinocytes), providing additional morphological contrast accurately co-registered within the RSOM field of view.

Another example of the multi-scale capability and the contrast complementarity of IMMSOM was presented by the hybrid imaging of a wildtype zebrafish larva, concurrently visualizing several anatomical structures such as melanophores, myomeres and vertical myosepta. Through the use of mesoscopic and microscopic optoacoustic imaging modalities, we were able to reveal the global pigment distribution in the entire fish, as well as structural details of single melanophores in a selected region of interest. Thus, IMMSOM offers the capability to simultaneously study pigment formation in zebrafish at the cellular and whole-organism level, while relating those processes to other anatomical structures at optical-diffraction limited resolution.

Overall, the hybrid system presented herein offers great potential for developmental biology studies, providing a comprehensive view of different complementary anatomical features at different scales without the necessity of staining, whereas RSOM facilitates high-resolution imaging beyond the optical diffusion limit. These capabilities are expected to enhance our understanding of morphological processes, simultaneously capturing cellular, tissue and organ level organization in developing organisms^1^.

At the current stage, the transmission mode configuration of IMMSOM limits its potential *in vivo* applicability to small organisms or flat specimens, such as mouse ear or tail. An implementation of the system in reflection mode could overcome these limitations. A first example of reflection mode multi-modal microscopy including OM can be found in^22^. Moreover, the acquisition speed of the OM modality could be improved by employing the existing galvanometric mirrors for beam scanning in a future configuration. Finally, the addition of several excitation wavelengths might further improve the differentiation of various tissue structures featuring characteristic absorption spectra.

## Methods

### Experimental setup

The optoacoustic modalities employ a pulsed diode-pumped solid-state laser (Flare HP PQ Green 2k 500, Innolight GmbH, Hannover, Germany; energy per pulse: 570 μJ, pulse width: 1.8 ns, repetition rate: 1.2 kHz, M^2^: ~1.3) to irradiate the sample at 515 nm. A flip mount mirror is used for the coupling of the laser beam into a properly modified inverted optical microscope (AxioObserver.D1, Zeiss, Jena, Germany). For optoacoustic microscopy, the laser beam is attenuated by a combination of neutral density filters in order to reduce the pulse energy at the focus. A 25 μm pinhole is used for spatial filtering while the beam is expanded to a size bigger than the back aperture of the employed objective lens (Plan Apochromat 10X, Zeiss, Jena, Germany; air immersion, NA: 0.45). Thus, only the inner part of the beam is focused into the sample, whereas the outer part is blocked by an iris aperture, resulting in a cleaner diffraction limited focusing. The sample is placed on a 170 μm thick glass bottom petri dish above the objective lens. It is filled with water in order to provide efficient acoustic coupling of the sample to the ultrasound detector. The spherically focused transducer (SONAXIS, Besancon, France; bandwidth: approximately 25–125 MHz, focal distance: 1.8 mm, F/D: ~1), which is positioned above the sample, has a central frequency of 78 MHz and is aligned coaxially and confocally with respect to the illumination. Prior to every measurement, the alignment is performed by focusing the laser onto a thin spot of black varnish next to the sample and maximizing the signal amplitude while positioning the transducer in three dimensions. The detected broadband acoustic signals are amplified using a low noise amplifier (AU 1291, Miteq, New York, USA; gain: 63 dB) and recorded via a high-speed 12 bit digitizer (Compuscope EON 121 G20, Gage Applied, Lockport, USA; max. sampling rate per channel: 1 GS/s). The data acquisition is triggered by a fast photodiode (DET36A, Thorlabs, Newton, NJ, USA), which detects scattered light at the laser output. In order to record an image, the sample is scanned step wise in the lateral direction by means of a high-precision motorized xy-stage (MLS203 2, Thorlabs) on top of the microscope, while the transducer and the illumination remain fixed. At each measurement position, the time-resolved optoacoustic signals are recorded and averaged twenty times to improve the SNR.

In raster-scan optoacoustic mesoscopy (RSOM), the 515 nm beam is focused by a lens below the sample holder such that the whole sample is illuminated by its opening cone. The lens and the objective lens for the microscopy modalities can be easily interchanged with an objective revolver. In contrast to OM, the sample remains fixed during the measurement whereas the transducer is raster scanned laterally above the sample using two motorized linear piezo stages (M 683.2U4, Physik Instrumente GmbH & Co. KG, Karlsruhe, Germany). For 3D imaging, the focal point of the transducer is placed slightly above the surface of the specimen. Vertical positioning of the transducer is achieved by a high-precision z-stage (M 501.1DG, Physik Instrumente GmbH & Co. KG). For both optoacoustic modalities, the data acquisition and scan control are performed with Matlab (Mathworks, Natick, MA).

As far as the three multi-photon microscopy modalities are concerned, we efficiently excite the respective non-linear optical processes (SHG, THG and TPEF) by employing an Yb-based solid-state femtosecond laser oscillator, emitting near infrared pulsed light at a central wavelength of 1043 nm (YBIX, Time-Bandwidth, Zurich, Switzerland; pulse width: 170 fs, output average power: 2.8 W, repetition rate: 84.4 MHz). The laser beam is initially attenuated through a proper combination of neutral density filters and subsequently collimated and reduced in its diameter by a two lens telescope system. Following this, the beam is guided onto a high-precision set of galvanometric mirrors (6215H, Cambridge Technology, Bedford, Massachusetts, USA), which is used to perform a fast raster scanning in the selected xy-plane of the examined specimen. Subsequently, the laser beam is reflected by a suitable dichroic mirror (DMSP805R, Thorlabs, Newton, New Jersey, USA), which is reflective at 1043 nm, expanded six times by a telescopic lens system, being in a typical 4f configuration, and finally coupled into the inverted microscope. Diffraction limited focusing is achieved by the same objective lens that is used for OM. The focal plane of each imaging session is selected via a high-resolution motorized piezoelectric z-stage (MZS500 E, Thorlabs), which is mounted together with the xy-stage on top of the microscope. The generated backscattered SHG or TPEF signals are collected in reflection mode, following an inverse path through the objective lens and the visibly transparent dichroic mirror. An appropriate narrow bandpass interference filter (FB520 10, Thorlabs) in the case of SHG and a longpass filter (FGL550, Thorlabs) for TPEF select the desired detection wavelength range before the signals are recorded via an ultra-sensitive photomultiplier tube (PMT) (H9305 03, Hamamatsu, Hamamatsu City, Japan). On the other hand, the primarily forward propagating THG radiation is collected in transmission mode by employing an extra detection channel, which consists of an aspheric condenser lens (ACL25416U, Thorlabs; air immersion, NA: 0.79), a UV coated focusing lens (LA4052-UV, Thorlabs), a colorglass filter (FGUV5, Thorlabs), highly transparent in the detected THG UV wavelength of ~348 nm, and finally a second identical PMT. The interchange between the ultrasound transducer and the THG channel for two consecutive measurements can be accurately achieved without any disturbance of the examined specimen. The digitization and acquisition of the generated multi-photon signals, as well as the control of the galvanometric mirrors, is accomplished by a 16 bit DAQ card (PCIe 6363, National Instruments, Austin, Texas, USA; max. sampling rate per channel: 1 MS/s). The brightfield observation of the specimen is performed via a CCD camera (AxioCam ICc 1, Zeiss, Jena, Germany). The synchronization of the multi-photon setup devices is fully controlled through custom-designed LabVIEW programs.

### Image reconstruction, co-registration and processing

In optoacoustic microscopy, no image reconstruction is required, since efficient acoustic signal generation is confined to the optical focal volume, which is much smaller than the acoustic focus of the transducer (~23 μm laterally and ~200 μm axially at the central frequency). The signals are bandpass filtered in the range of 25–125 MHz and the signal envelopes are calculated using the Hilbert transform.

On the contrary, RSOM detects out-of-focus signals as well, making tomographic image reconstruction necessary. For each measurement position, the signals are assumed to be detected by a point detector at the focus of the transducer and subsequently projected into the sensitivity field volume using 3D filtered backprojection. The sensitivity field of the detector is modeled as a hyperboloid with finite focus diameter and a Gaussian weighted lateral cross section. Prior to image reconstruction, the optoacoustic signals are bandpass filtered in the 25–125 MHz range. The 3D backprojection code is implemented in Matlab and runs on a graphics processing unit.

Co-registration between the microscopic modalities was achieved through the imaging of a suture phantom prior to the biological experiments. The individual offsets of the different modalities were corrected with respect to the brightfield image, which served as a reference.

The final processing of the recorded images for all modalities was performed in ImageJ.

### Spatial resolution characterization

The phantom for the spatial resolution characterization of the optoacoustic microscopy modality consisted of black polystyrene microspheres with 954 nm diameter (Polybead, Polysciences Inc., Warrington, Pennsylvania). The microspheres were first treated in an ultrasonic bath to prevent agglomerations and fixed in pure agar. A thin slice of the agar phantom was placed on a glass bottom dish, covered with ultrasound gel for acoustic coupling and sealed with a piece of plastic foil. The pulse energy at the sample was measured to be ~12 nJ. In order to estimate the lateral resolution of the system, the original profile of the microsphere was approximated to follow a Gaussian shape, with the nominal diameter corresponding to ±3σ. The measurement process was assumed to be a convolution of the microsphere profile with the Gaussian beam focus. Thus, the lateral resolution could be determined by evaluating the expression


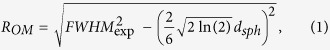


where FWHM_exp_ represents the measured FWHM of the microsphere and d_sph_ its actual diameter. For the resolution characterization, we used the raw optoacoustic signals without calculating the envelopes.

The lateral resolution of the multi-photon modalities was estimated through the TPEF imaging of 100 nm fluorescent spheres (TetraSpeck Fluorescent Microspheres Size Kit, Invitrogen, Carlsbad, California, USA), acting as point like signal sources and essentially representing an xy-view of the point spread function (PSF) in the final image. On the other hand, the respective axial resolution was determined by performing a THG z-scan with 500 nm steps, using a standard 170 μm coverslip (No. 1.5, Paul Marienfeld GmbH & Co. KG, Lauda Königshofen, Germany) for the efficient non-linear signal excitation at the upper glass-air optical interface. In both cases, the pulse energy at the focal plane was estimated to be ~1.3 nJ.

### Hybrid mouse ear imaging

The excised mouse ear was embedded in pure agar inside a glass bottom dish. The FOV of the RSOM measurement was 2 mm × 2 mm, while the scanning step size of the transducer was 3 μm. The acquisition time was ~6 min. We performed two reconstructions, using the full detection bandwidth of 25–125 MHz in one case and the upper half of the bandwidth, 75–125 MHz, in the other case. For the OM measurement, the sample was scanned in 1.8 μm steps in a region of 375 μm × 375 μm within ~80 min. To improve the SNR, the signals were averaged forty times and the pulse energy at the sample was increased to ~85 nJ.

The multi-photon images were recorded in the same region as the OM scan through a depth of 50 μm in 2 μm vertical steps and averaged twenty times for SNR improvement. A single averaged image was acquired in ~30 s in all multi-photon modalities. The pixel size for each image was 508 nm, while the pulse energy at the sample was ~1.3 nJ.

### Hybrid zebrafish larva imaging

The larva was placed on a glass bottom dish, covered with ultrasound gel and sealed with a piece of plastic foil. The RSOM measurement was performed in a scanning region of 4 mm × 4 mm with ~10 min scanning time and 5 μm step size of the transducer. The subsequent OM scan was performed in a FOV of 315 μm × 315 μm with an acquisition time of ~65 min. Because melanin is a strong absorber in the visible range, the energy at the sample was reduced to ~8 nJ in order to prevent damage to the specimen.

For the multi-photon measurements, the pulse energy of the fs laser had also to be reduced down to ~0.5 nJ in order to avoid photodamage effects due to the strong absorption of melanin. The SHG and THG images were recorded following the averaging of thirty frames for SNR enhancement, while pixel size and acquisition time were similar to the mouse ear imaging session. Saturated pixels were removed in order to improve the visibility of the respective images.

No live specimens were used in the experiments.

## Additional Information

**How to cite this article**: Soliman, D. *et al*. Combining microscopy with mesoscopy using optical and optoacoustic label-free modes. *Sci. Rep*. **5**, 12902; doi: 10.1038/srep12902 (2015).

## Figures and Tables

**Figure 1:**
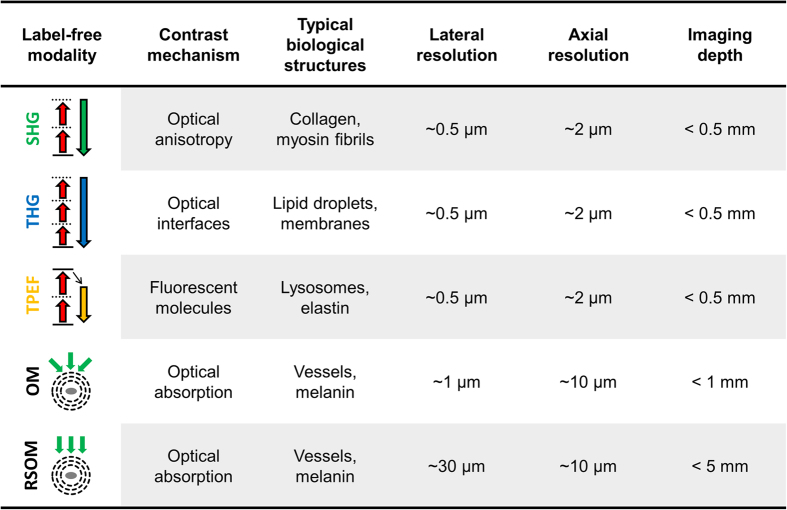
Comparison of the different label-free imaging modalities combined in the hybrid device.
